# An Interactive Tool for Animating Biology, and Its Use in Spatial and Temporal Modeling of a Cancerous Tumor and Its Microenvironment

**DOI:** 10.1371/journal.pone.0133484

**Published:** 2015-07-20

**Authors:** Naamah Bloch, Guy Weiss, Smadar Szekely, David Harel

**Affiliations:** Dept. of Computer Science and Applied Mathematics, Weizmann Institute of Science, Rehovot, Israel; Jacobs University Bremen, GERMANY

## Abstract

The ability to visualize the ongoing events of a computational model of biology is critical, both in order to see the dynamics of the biological system in action and to enable interaction with the model from which one can observe the resulting behavior. To this end, we have built a new interactive animation tool, *SimuLife*, for visualizing reactive models of cellular biology. SimuLife is web-based, and is freely accessible at *http://simulife.weizmann.ac.il/*. We have used SimuLife to animate a model that describes the development of a cancerous tumor, based on the individual components of the system and its environment. This has helped in understanding the dynamics of the tumor and its surrounding blood vessels, and in verifying the behavior, fine-tuning the model accordingly, and learning in which way different factors affect the tumor.

## Introduction

The modeling of biological systems by computerized models that support interactive executions (simulations) provides the opportunity to integrate a large amount of experimental data, and generate a comprehensive overview of the system as a whole. This includes the ability to observe the dynamics in operation, by an abstracted animation of the model, which is essential for a clear understanding of the biology represented in the model and is convenient as a basis for further analysis.

Systems biology, mathematical modeling and computational approaches can make important contributions to research and development in biology [1,2], and these are, in fact, becoming increasingly important in efforts to better understand complex biological behaviors. Extensive attempts to model and analyze biological systems or processes have been carried out [3,4], for the most part by traditional mathematical modeling [5–19], using a top-down approach, whereby the known behavior of the system is built into the model. A different approach, which has been termed *executable biology* [20] focuses on the design of fully executable models that mimic complex biological phenomena, and is usually done bottom-up [21–25]. For a review see [2]. Another familiar example of biological system modeling includes the blue brain project to study the brain's architectural and functional principles [26]. There, the system’s dynamics emerge from the model via reverse engineering using the NEURON software together with a biologically realistic model of neurons, based on exact mathematical representation.

Although computational models usually contain a lot of essential details, in most cases they cannot pass on to the user one of the most important aspects of the system being modeled, which is actually seeing it in operation [27]. Visualization is an effective way of representing the dynamics of a model. This should include at least the system’s components, their interactions and the effect of changes to parameter values. The technique of *reactive animation* (RA), whereby the model of the reacting system is connected smoothly to an animation tool [28–30], has been used in the past to successfully model several nontrivial biological systems [22–24]. In [24] the dynamic architecture of a lymph node was modeled. RA aided in observing the behavior leading up to the unique meeting between the specific T and B cells, or how they can miss each other at times depending on the other factors present. In [23] the development of the mammalian pancreas was modeled. Here RA aided in observing the physical 3D formation of the pancreas as well as seeing which cells it is composed of at each stage. Also, changing the layout of the blood vessels revealed shapes that are different in nature from the genuine pancreatic structure. In [22] the differentiation of T cells in the thymus was modeled. RA revealed a previously unknown existence of competition among thymocytes for space and stimulation, which is essential for generating the normal structure and function of the thymus organ.

In this paper we describe *SimuLife*, a new RA-based interactive tool for animating computational models of cellular biology. Our main goal was to make the tool generic, so that it could serve a wide variety of types of biological systems. We also wanted SimuLife to help improve the experience of both developers and users, and to give a realistic look to the running model so that it would be accessible to biologists, and even laypeople. With this in mind, we have endowed the tool with a user-friendly interface that allows one to easily manipulate and experiment with the parameters, as well as with other aspects of the tool, such as the image types used, their colors, what to include in a view, etc.

The advantage of SimuLife, and one of the things that make the tool so attractive, is that it is accessible directly via the web and does not require any specific downloads or installations (*http*:*//simulife*.*weizmann*.*ac*.*il/*). In addition its graphics is 3D allowing one to intuitively follow the morphological arrangement.

As mentioned, the main purpose of creating this tool was to constitute a first step towards a generic tool that would eventually be able to support a multitude of different kinds of biological models. Nevertheless, the process of building the tool was driven by our work on a complex computational model of a cancerous tumor and its microenvironment, which was done in parallel. In the present paper we illustrate the use of the tool and its capabilities on this particular model, and in future work we plan to adapt SimuLife to other types of systems too.

Cancer research is of great importance. It refers to many different and distinctive diseases, all of which stem from the same state of abnormal growth and regulation of cells, which proliferate in an uncontrolled way. Amongst the hallmarks of a cancerous cell, such as those stated above, it has been revealed that cells around the tumor constitute what is termed the tumor microenvironment [31]. The cancerous tumor cannot survive or progress on its own; it depends on the dynamic microenvironment in which it originates and the bi-directional interactions with this surrounding. The bi-directional cross-talk between the tumor and its environment takes place either by secretion of signals or by cell-cell interactions. This communication is very important and can act to enhance or block tumor formation.

Angiogenesis is the process of new blood vessels growing from pre-existing ones. It is a normal and vital process in embryo development and wound healing, but is also a fundamental step in the transition of tumors from a dormant state to a malignant one. Without blood vessels, tumors cannot grow beyond the size of 1mm^3^ [32]; they need a large supply of oxygen and nutrients delivered to their cells. When the tumor is under hypoxia (lack of oxygen) [33] a series of events occurs, a crucial one being the secretion of VEGF from the tumor cells, leading to angiogenesis.

Using the SimuLife tool, we can visualize the ongoing events of the cancer model, including angiogenesis and its effect. This is of great importance, as it enables one to see the development and morphology of the tumor and its surroundings, understand the dynamics of the system based on its individual components, verify the behavior, fine-tune the model accordingly, and manipulate the input in order to visualize its effect on the resulting morphology. The latter includes changing parameters to see what happens to the tumor either without angiogenesis or when the level of factors affecting angiogenesis is changed.

## Results

### Tool development

An interactive animation tool, *SimuLife*, for visualizing models of cellular biology, was built. SimuLife continuously receives inputs from the reactive model and draws and updates the graphics based on the changes. The main principles we used in designing and building SimuLife were as follows; To make it fast, efficient, able to support thousands of objects, use realistic-looking images, show good performance, be as generic as possible, work both in real-time and offline using a pre-recorded file, present the animation in 3D, be interactive and with a user-friendly interface, and be web-based. We wanted to render it free from having to download or install specific tools, not require one to purchase a license or to have to use a propriety language.

**Fig 1:**
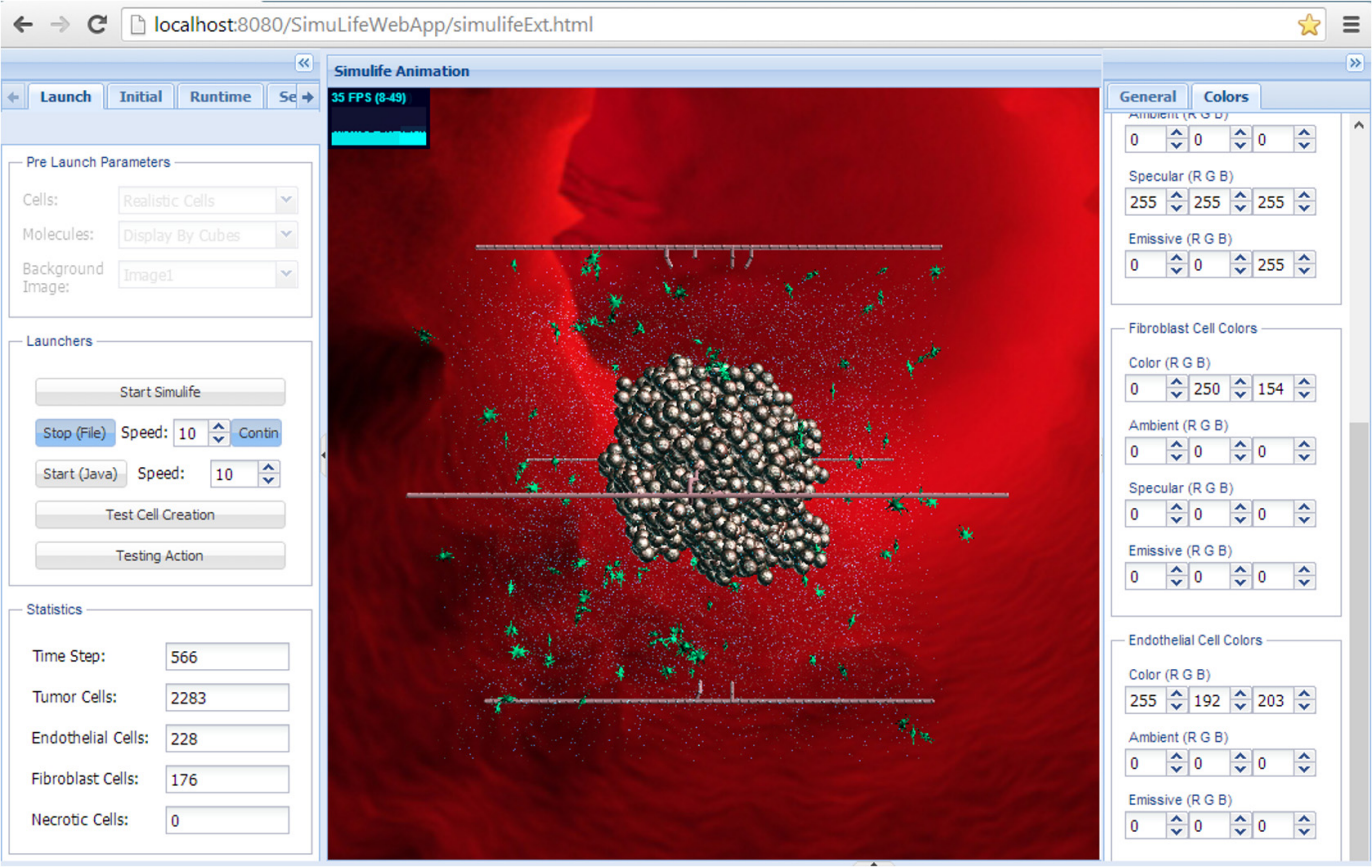
SimuLife interface. The middle window is the animation screen. On the sides are tabs with various options for running the model and changing elements in the animation.

SimuLife’s GUI contains a main screen showing the actual animation, and side tabs with various options for the computational model itself and the animation images (Fig 1). It also provides statistics regarding the current time step and amount of objects, which are updated throughout the run (see demonstration in S1 Video or at *http*:*//youtu*.*be/xsOXtD7-LjE*).

SimuLife has the following capabilities (see demonstration in S2 Video or at *http*:*//youtu*.*be/_U9rw1ACGhM*): Examples referred to are taken from the cancer model.

**Fig 2:**
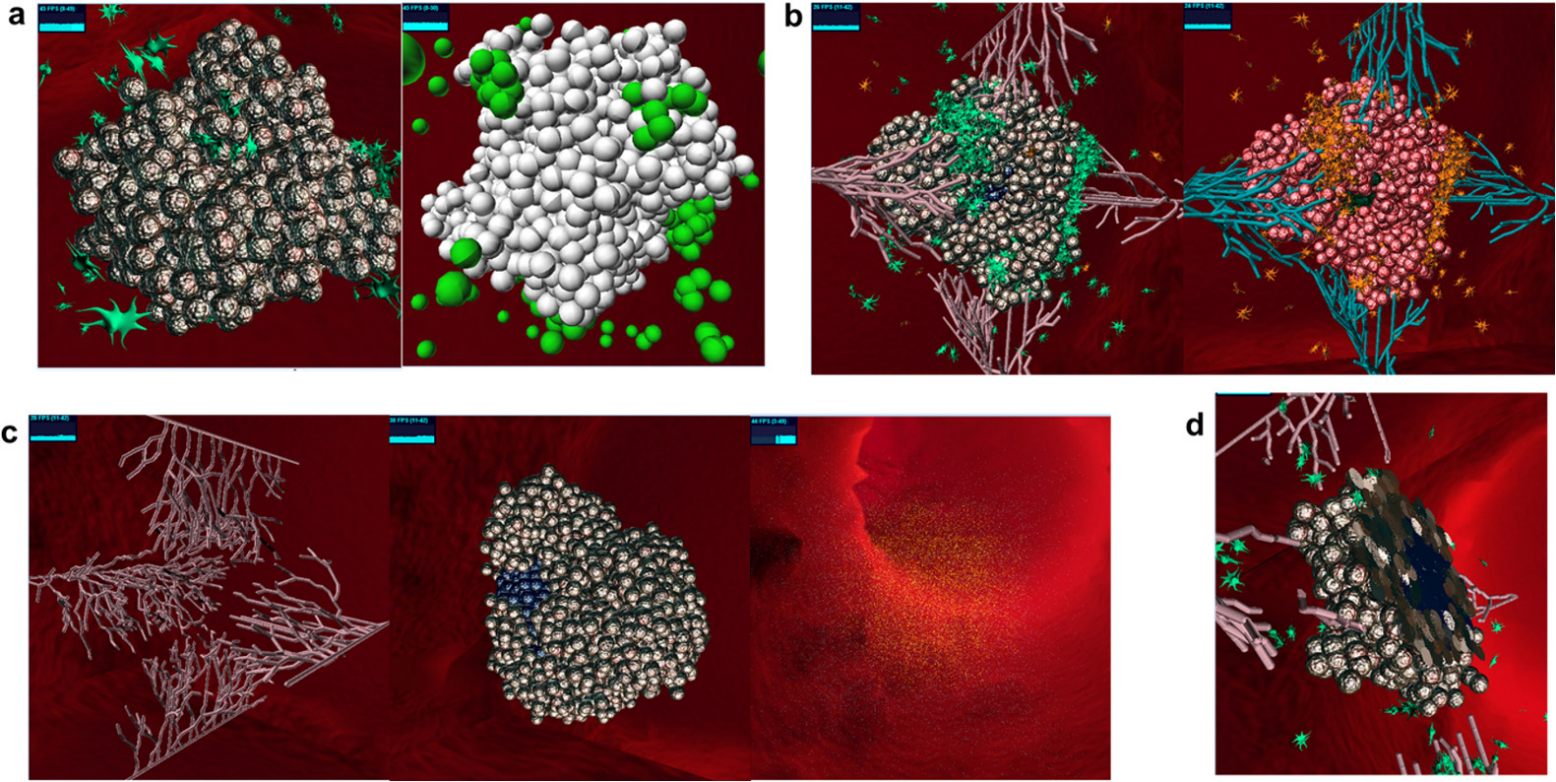
SimuLife images, presenting different capabilities. (a) Can use more realistic images (left) or simple spherical images (right). (b) Default colors (left) or a possible change of colors (right). (c) Make certain objects invisible in order to focus on other ones. Left – blood vessels, center – tumor, right – molecules. (d) Slicing: a 2D cross section (in this case of a tumor, showing the inside core).

- Represent cells using either simple spherical images or more realistic-looking ones (the latter is more costly computationally and can affect the performance) (Fig 2A).
- Represent molecules by density distribution, at each radius or by cubes.
- Change general or specific parameter values prior to or during a run (see demonstration in S3 Video or at *http*:*//youtu*.*be/bCkujp1E3m0*).
- Click-and-select individual visible objects, or select hidden objects (such as those inside the tumor) by choosing from a list, and receive relevant information about them from the model (position, parent cell, etc.).
- Create or kill objects, either specific ones or at random (see demonstration in S4 Video or at *http*:*//youtu*.*be/Khgej9Cs0jo*).
- Change the colors of the images (Fig 2B).
- Make certain objects invisible in order to focus on other objects (Fig 2C).
- Slice the animation at a given point along any of the 3D axes, in order to view a 2D cross section (Fig 2D).
- Zoom in and out, spin the animation around the center, and navigate to any 3D position.
- Use a pre-recorded file of a run, in order to see the results faster and/or later.
- Play with the speed in which the file is read, and hence with the speed of the animation, and pause it at any given time.

### Using SimuLife to animate a computational model of a tumor

The advantages of such a tool are vast; whilst the computational model holds the information for each of the individual objects, SimuLife allows one to visualize the information of all the objects together at once. Although SimuLife was developed to eventually serve as a generic tool, one that will be able to connect to many different kinds of biological models, it is currently used for a specific biological model – a Statecharts-based spatial and temporal model of a cancerous tumor and its microenvironment, developed using the Rhapsody tool from IBM (*www*.*ibm*.*com/software/awdtools/rhapsody/*).

Our model focuses on a three dimensional tumor, going from a single cancerous cell through the formation of a primary avascular tumor, via the secretion of angiogenic factors and recruitment of nearby blood vessels, to a completely vascularized tumor. Tumor cells and endothelial cells (cells of the blood vessels) were modeled with regard to their size, position, state, proliferation, input from the surrounding area and output into the surrounding. Using the Statecharts language (see materials and methods section), a generic behavior was modeled for each kind of object, and during the executions of the model many instances of the objects were generated to represent each specific one taking on its explicit states accordingly. Many parameters were used in the model, such as cell size, area size, oxygen threshold, VEGF threshold, rate of proliferation, secretion rate, and more. The values of these parameters can easily be changed to see the effect on the system of any one parameter or of a combination thereof. Values of parameters used in the model do not have units of measurements, but are quantified relative to each other. In this way qualitative comparisons can be made with biological experiments. Nonetheless, many of the general parameters such as time, size, amounts, have approximate equivalent real values to them and thus allow for quantitative comparisons to real tumor growth dynamics. More details on the model and a set of parameters and their values can be found in (S1 Text). The result is a comprehensive, dynamic, reactive, 3D spatial and temporal computational model of a cancerous tumor growth and its microenvironment.

Analyzing the behavior of the model is a vital step for understanding the dynamics of the system, and for being able to compare it to existing biological data in order to check the model’s correctness.

**Fig 3:**
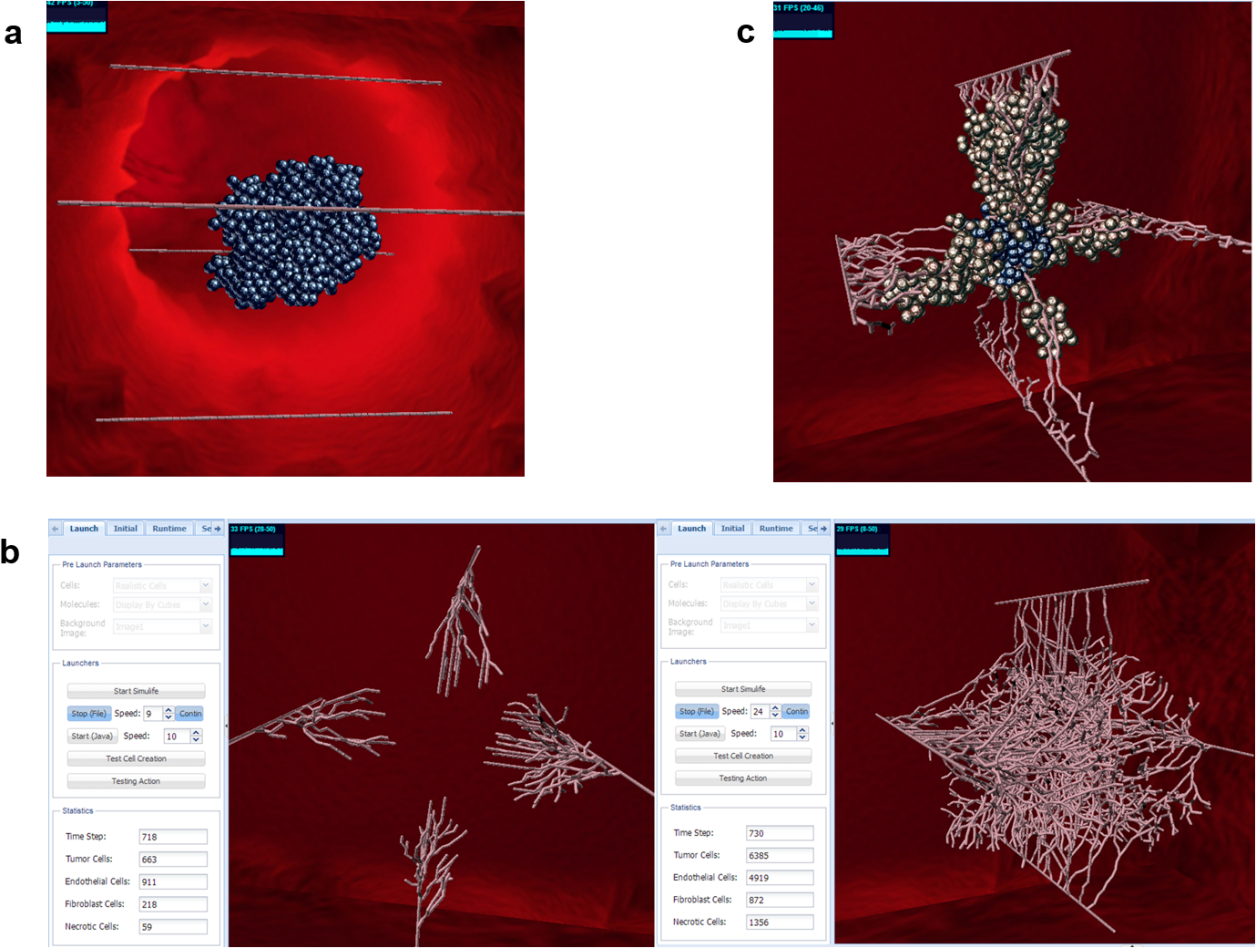
SimuLife images for results of different cases that were tested in the model. (a) No angiogenesis. Tumor does not develop (necrotic (dead) cells are in blue). (b) High vs. low VEGF secretion simulations. Left for low VEGF, right for high VEGF, with the amounts appearing in the tabs at the left of the image. Both images are presented at approximately the same time step – at low VEGF angiogenesis has only begun, whereas at high VEGF there are many activated and branched vessels. (c) The tumor growing outward, towards the blood vessels, in the case of a small number of vessels, due to defective vessel growth.

In the cancer model, we used SimuLife in order to understand the system better, compare to known biological data, verify that the model does not possess non-acceptable biological behaviors, fine-tune various parameters of the model, and test new and interesting possibilities. Some, of many examples of this include:

a) Testing the system under conditions of no angiogenesis (i.e., no blood vessels that grow towards the tumor). Without the tumor’s microenvironment, which includes angiogenic blood vessels, the tumor cannot survive, due to low oxygen/nutrient supply [33–36]. Therefore, as expected, this resulted in a primary tumor that stopped growing at some point, and then started to die off (Fig 3A). This could have been achieved by cell population curves of active cells, necrotic cells, endothelic cells and perhaps oxygen and VEGF curves. However, visualization shows the dynamics of all of the cells and molecules together, and allows one to get a sense of how it happens – which cells are killed off first, the size the tumor reaches, etc.

b) Testing the effects of high vs. low VEGF secretion. VEGF secretion by the tumor cells is what recruits the vessels towards the tumor in order to supply it with oxygen/nutrients [35]. The lower the VEGF level, or the longer it takes to reach the blood vessels, the longer it will take the endothelial cells to become activated to form the angiogenic network. Playing with this parameter reveals that low VEGF secretion results in far fewer blood vessels, whilst using high VEGF secretion values results in many blood vessels already being formed at the same time point (Fig 3B). Here too, visualization aids in seeing not only the amount of endothelial cells that are created over time but also the morphology that the blood vessels form at different time stages, as they follow the VEGF gradient to reach the tumor. In addition, it is possible to view the change in the VEGF gradient throughout space.

c) Testing defective blood vessel growth. This is not taken from the literature but instead is an example showing the shape that the tumor takes on in different cases. Here the blood vessels were not allowed to branch into new ones, but only to become longer, so there is no exponential growth of vessels, hence less oxygen. This caused the tumor to grow towards the oxygen source, “hugging” the blood vessels and thus forming a branching shape and not a sphere-like shape (Fig 3C). This different growth pattern of the tumor was revealed with the aid of the SimuLife tool.

## Materials and Methods

**Fig 4:**
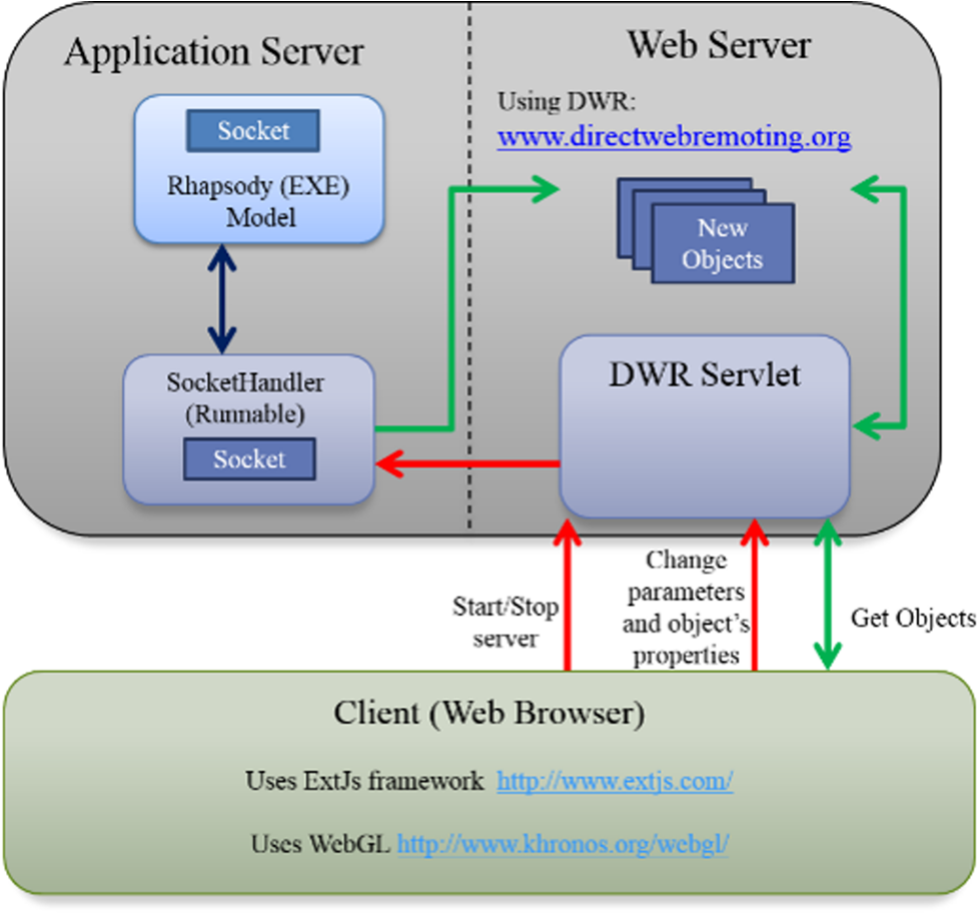
The architecture of SimuLife. Composed of a client side and a server side. The client side is a web browser presenting graphically the output of the model executed. The server side is divided into the application server that maintains the executed model, and a web server that creates the new objects that are sent to the web browser, according to the information obtained from the model. Information can also be sent from the client side (the user interface) to the model via the web server.

SimuLife is an interactive animation tool that interacts with the computational model, sending information to it and receiving information from it (both as XML files). This is in the spirit of the *reactive animation* technique [28–30]. SimuLife then draws and/or modifies the graphics based on the changes in the model. SimuLife is based on WebGL (Web Graphics Library), and a JavaScript API (THREE.js framework in our case) for rendering interactive 3D graphics within any compatible web browser without the use of plug-ins. The client side is Chrome and the communication with external engines is carried out via sockets (Fig 4). The bases for the realistic versions of the images (e.g., cells) were prepared by a professional animator and are in COLLADA, a format that can be used with our framework. SimuLife is open source and the scripts used to build it are available at https://github.com/simulife/simulife.

Some of the challenges arising during the tools’ development include:

- Dealing with animating thousands of behavior-rich objects, while trying not to impair performance. One of the ways we did this was by grouping many objects together into a single object and ungrouping when necessary. Each object was treated individually and was referred to separately when needed, but when not in use, e.g., when inside the tumor or on a side not visible to the user, the objects were grouped in order to take up less computer memory so that the performance will not be harmed.
- When showing a slicing of the animation, with its 2D cross section, there was a need to make the sliced area appear to be flat, meaning that each of the images in the border had to be “cut”. 3D objects are hollow and when slicing them there is a need to close the unclosed sliced 3D shape. In order to solve this we used ThreeBSP package, a CSG plugin for Three.js (https://github.com/sshirokov/ThreeBSP), which allows us to subtract two 3D objects from each other; the 3D area that is to be cut from the scene at each of the three axes was subtracted from the 3D cell, resulting in a closed sliced image.
- Presenting a large amount of objects (such as millions of molecules). We solved this by presenting the gradient distribution of the molecules instead of each of the individual molecules. This was done by displaying the total amount of molecules either at every sphere from the center of the tumor or at every cube throughout the space modeled.
- Rendering the connection between separate but adjacent objects to appear as a continuous object (such as presenting connecting endothelial cells as a blood vessel). This was done by joining the center of each object and displaying the connection by a tube-like image.

### The Statecharts modeling language

**Fig 5:**
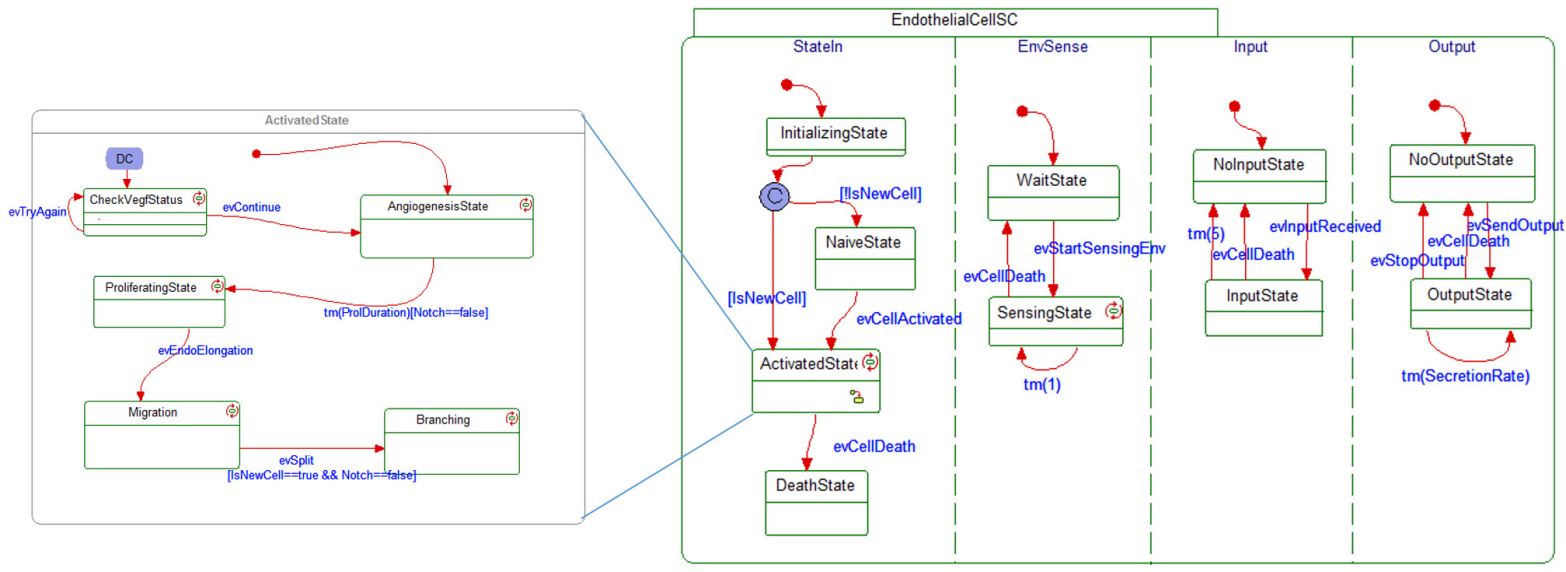
An example of a statechart used in the cancer model. A stereotype behavior was created for each of the objects using Statecharts. During execution of the model many instances of each object type are generated to represent every specific object taking on its explicit states accordingly. The statechart of the endothelial cell, as shown here, consists of various states that the cell can be in, some of which can exist in parallel (presented by the broken lines) and others that reside within other states, in a hierarchical fashion.

Our computational cancer model was designed using the language of Statecharts as the centerpiece [37,38]. Statecharts makes it possible to describe the behavior of reactive systems in a discrete fashion, using an edifice of states and the transitions between them with a mix of hierarchy and concurrency. In this way, one can specify the behavior of the individual entities that take part in the cancer process and insert biological data, in order to capture and be able to execute the dynamic behavior and morphology of the system. Statecharts are executable on several appropriate tools, such as Rhapsody, available from IBM, which is the tool we used (*www*.*ibm*.*com/software/awdtools/rhapsody/*) (see Fig 5).

## Discussion

In order to better understand biological models, a detailed and realistic visualization of the model can be very useful. For this purpose we developed the *SimuLife* tool, where an animation is dynamically constructed at real time according to the model, producing a different interactive visualization of the system’s run each time.

As mentioned, although visualization of biological models has been used in the past [22–24], one of our goals was to make the tool generic, so that it could serve a wide variety of types of biological systems, especially those describing cells and using agent-based modeling. In principle, any model can be connected to SimuLife, as long as it can output messages to the animation via a socket. This will involve making changes to the interface and the images used according to the specific model. We also wanted SimuLife to help improve the experience of both developers and users, and to give a realistic look to the running model so that it would be attractive and useful to biologists, and even to laypeople.

In our research we used the language of Statecharts with the Rhapsody tool in order to create a comprehensive 3D model of a cancerous solid tumor, together with its microenvironment. SimuLife aided in tracking and validating the development and progression of the tumor and the vessels. It enabled seeing the tumor cells at their precise 3D locations, together with the blood vessels that consist of the individual endothelial cells, and their elongation towards the tumor, as well as distribution of molecules’ density, and more. SimuLife allows to easily play with the animation, send commands back to the model during runtime and observe the immediate resulting output, as well as adjust many aspects of the animation itself to meet the user’s requirements. It also enables viewing only certain elements of interest and getting real current quantitative data. Such a tool is important especially in the case where the spatial organization is of major interest. Using SimuLife for the cancer model enabled us to see how the model resembles the behavior of a solid tumor; a necrotic core developed in the inner part of the tumor [39] and the branching of the blood vessels occurred more often as they approached the tumor [40]. These and more are behaviors that emerged from the model and were revealed by the animation. We also came to the conclusion that the tumor has a turning point, where it either dies or recovers. This was revealed by varying the values of key parameters that affect amounts of VEGF and oxygen and noting the behavior of the tumor together with the rest of the system’s components. These results will be discussed more thoroughly in a follow-up paper, in which we will describe in more detail the biological issues, and the relevant insights gained from the interactive visualization of the cancer model.

Other visualization tools that describe computational models of cancer exist [41,42]. However, they are fundamentally different from SimuLife in that SimuLife is based on reactive animation, where the animation platform is an entity totally separate from the model; whilst the aforementioned tools are the actual models, SimuLife is solely the animation driven by the model, built using another generic approach, Statecharts, and its underlying tool Rhapsody, and which is interlinked with SimuLife via RA. By separating these two facets, each can be constructed using state-of-the-art tools [29].

With more effort we aim to continue improving the SimuLife tool, making it more generic—able to easily connect to other biological models, other modeling engines, or other programming languages, as well as improving its performance. Finally we are continuing to work on the cancer model with the aid of the SimuLife in order to learn more about the tumor and perhaps gain new insights.

## Supporting Information

S1 Text Cancer model details and a list of parameters. Details regarding the cancer model that was used with the SimuLife tool, together with a list of the parameters used in the model and their default values.
(DOCX)

S1 Video A full run of the cancer model in SimuLife. (MP4)

S2 Video SimuLife tool capabilities. (MP4)

S3 Video Changing parameters of a model in SimuLife. (MP4)

S4 Video Killing/creating cells of a model in Simulife (MP4)
