# Supplementary material for: An Interactive Tool for Animating Biology, and Its Use in Spatial and Temporal Modeling of a Cancerous Tumor and Its Microenvironment
Source: PLoS One. 2015 Jul 20;10(7):e0133484. doi: 10.1371/journal.pone.0133484 (PMC4508114; doi:10.1371/journal.pone.0133484)
Supplement: S1 Text — Details regarding the cancer model that was used with the SimuLife tool, together with a list of the parameters used in the model and their default values. (DOCX) [file pone.0133484.s001.docx]

**An Interactive Tool for Animating Biology, and Its Use in Spatial and Temporal Modeling of a Cancerous Tumor and Its Microenvironment**

**Naamah Bloch*, Guy Weiss, Smadar Szekely, David Harel
Dept. of Computer Science and Applied Mathematics, Weizmann Institute of Science, Rehovot, Israel**

**Supporting Information:**

**The Cancer model**

The model consists of a closed “world” that defines the system – the tumor and its microenvironment. The model begins with a single cell, which at some stage mutates to become cancerous, and then starts to proliferate. Gradually, a primary tumor develops out of the bulk of cancerous cells that originated from the single mutated cell. Each cell has its specific 3D position within this world and takes on its own behavior, depending on its surroundings. The tumor cell constantly senses several elements from its immediate surroundings such as oxygen and growth factors. Since a cell needs oxygen in order to survive, it continuously consumes available oxygen at a certain level. If the amount of oxygen is insufficient, the cell will not be able to proliferate, will go into a state of hypoxia and will start secreting angiogenic factors (VEGF). The VEGF is secreted from the specific cell in pulses, and continues until the cell is able to consume enough oxygen so as not to be in a state of hypoxia. The VEGF diffuses out of the cell in a random movement fashion. If the absence of sufficient oxygen continues, the cell enters a state of anoxia and becomes necrotic and no longer active.

The model also begins with a number of initial blood vessels located either around the border of the defined world, or at random positions. These vessels are made up of many individual endothelial cells. The endothelial cells are of the same size as the tumor cells, and similarly sense their environment constantly. They also possess receptors for VEGF; hence once they bind to an amount of VEGF above a specified threshold within a certain time, they become activated and begin the process of angiogenesis, whilst the bound VEGF is eliminated. Angiogenesis involves elongation of the blood vessel in a direction that follows the VEGF gradient. This occurs by proliferation of those endothelial cells that have become activated. This will continue as long as the endothelial cell continues to bind to enough VEGF for its continuation, or if it has not encountered another endothelial cell, in which case it will join it and stop elongating. Activated endothelial cells can also split and branch out of their main vessel if they indeed bind to a high amount of VEGF in a short amount of time. Due to the delta-notch inhibition between adjacent cells, once an endothelial cell is activated, the neighboring cells cannot become activated too. Each endothelial cell secretes oxygen at a constant rate. Oxygen, like the VEGF molecule, diffuses out of the cell in a random movement fashion and disappears when consumed.

In this way, the newly produced blood vessels make their way to the tumor, and as a result the tumor continues to grow. This forms a unique spatial organization of the blood vessels as a consequence of the circumstances.

A minimal number of initial fibroblast cells, which are the main components of the ECM, are initially placed randomly around the tumor. During the model’s execution, those that are close to the tumor cells and have enough oxygen have a greater chance of becoming activated into CAFs (cancer associated fibroblasts). CAFs secrete VEGF in correlation with their hypoxia state, which helps recruit the blood vessels, and HGF (Hepatocyte Growth Factor), which helps the tumor cells proliferate. They also degrade the ECM around them, which helps tumor cells move. CAFs themselves have a much more motile phenotype and generally move towards the tumor. They do this by following the gradient of FGF (Fibroblast Growth Factor) that is secreted by the tumor cells.

**List of Parameters**

| **Parameter** | **Description** | **Default value** |
| --- | --- | --- |
| Hypoxia Level | Number of time steps that the cell does not have sufficient oxygen and starts secreting VEGF | 25 |
| Anoxia Level | Number of time steps that the cell does not have sufficient oxygen and dies | 1000 |
| Oxygen Consumption | The amount of oxygen a cell consumes every time step | 80 |
| Cell Size | The number of pixles to the power of 3 that each cell occupies | 2 |
| VEGF Secretion Amount | Number of VEGF’s secreted in each pulse | 25 |
| FGF Secretion Amount | Number of FGF’s secreted in each pulse | 100 |
| Duration For Summing HGF’s | The number of time steps back that sum up HGF amount | 8 |
| VEGF level for angiogenesis | Minimum VEGF level to initiate angiogenesis | 13 |
| Endothelial sensing radius | The radius that the cell checks around it for VEGF’s | 3 |
| Min VEGF for survival | The minimum amount of VEGF needed for survival of the endothelial cell | 1 |
| Oxygen Secretion Amount | Number of Oxygen’s secreted in each pulse | 100 |
| VEGF Summing Duration | The number of time steps back that sum up VEGF amount | 8 |
| Initial Vessels Growth | The way in which the initial vessels will grow | 1 |
| Duration for summing FGF’s | The number of time steps back that sum up FGF amount | 8 |
| Amount HGF secreted | Number of HGF’s secreted in each pulse | 10 |
| Fibroblast sensing radius | The radius that the cell checks around it for tumor cells | 5 |
| Probability to become a CAF | The probability for a normal fibroblast to become a cancer associated fibroblast | 0.02 |
| Model World Size | Pixles to the power of 3 that determine the full size of the world modeled | 100 |
| Initial oxygen level | The initial amount of oxygen in each pixel | 10 |
| Initial vessels | The number of initial vessels | 4 |
| Oxygen moving distance | The number of pixles the oxygen will move every step | 1 |
| Proliferation duration | Time it takes for cell to proliferate | 15 |
| VEGF moving distance | The number of pixles the vegf will move every step | 2 |
| FGF moving distance | The number of pixles the fgf will move every step | 1 |
| HGF moving distance | The number of pixles the hgf will move every step | 1 |
| Oxygen Move Rate | The time in which the oxygen makes one move | 3 |
| VEGF Move Rate | The time in which the Vegf makes one move | 2 |
| FGF Move Rate | The time in which the fgf makes one move | 2 |
| HGF Move Rate | The time in which the hgf makes one move | 2 |
| Secretion Rate | The time for one pulse of VEGF/oxygen to be secreted | 10 |
| Number of initial fibroblasts | Number of initial fibroblasts | 50 |
| Probability to become cancerous | Initial Probability for a normal cell to become cancerous | 0.2 |
| Probability to proliferate | Initial Probability for a cell to proliferate at a given moment | 0.8 |
| Probability to bind | Initial Probability for a cell to bind a molecule | 0.8 |
| Simulation time | Duration of simulation run | 1500 |

*****These parameters do not have units of measurements, but are quantified relative to each other.

The following have approximate equivalent real values to them as described:

Each time step in the model is approximately equal to 1-2 hours

Each 10^3^ cells in the model are approximately equal to 100^3^ cells

Each position (pixel) in the model is 5µm

Every VEGF molecule in the model represents ~10 real VEGF molecules

Every oxygen molecule in the model represents ~7 million real oxygen molecules
